# Role of Colony Stimulating Factor 1 (CSF-1) and Its Receptor CSF1R: Macrophage Repolarization for Glioblastoma Treatment

**DOI:** 10.1021/acsptsci.5c00007

**Published:** 2025-09-12

**Authors:** Gaurisha alias Resha Ramnath Naik, Rachana S P, Sandesh Ramchandra Jadhav, Rahul Pokale, Paniz Hedayat, Deepanjan Datta, Bhupendra Prajapati, Srinivas Mutalik, Namdev Dhas

**Affiliations:** † Department of Pharmaceutics, Manipal College of Pharmaceutical Sciences, Manipal Academy of Higher Education (MAHE), Manipal, Udupi, Karnataka State 576104, India; ‡ Centre for Research Impact & Outcome, Chitkara College of Pharmacy, Chitkara University, Rajpura, 140401 Punjab, India; § Faculty of Pharmacy, Silpokorn University, Nakhorn Pathom 73000, Thailand

**Keywords:** glioblastoma multiforme (GBM), tumor-associated macrophages (TAMs), colony stimulating factor-1 (CSF-1), macrophage polarization, CSF1R signaling pathways, immunotherapy

## Abstract

Glioblastoma multiforme (GBM) is the most aggressive and prevailing form of primary brain tumor, illustrated by its rapid growth and invasive nature. GBM continues to be highly incurable despite advancements in treatment due to its complex tumor microenvironment (TME) and the unique characteristics of tumor-associated macrophages (TAMs). This review explores the function of macrophages within the TME of GBM, specifically emphasizing the impact of colony-stimulating Factor-1 (CSF-1) and its receptor CSF1R in macrophage biology. The progression, survival, and differentiation of TAMs, which often rely on immunosuppressive properties that contribute to tumor growth and treatment resistance, are facilitated by elevated CSF-1 levels in GBM. The inhibition of CSF1R presents a promising therapeutic strategy, as it selectively targets tumor-promoting macrophages while sparing antitumor macrophages. Preclinical evidence demonstrates improved survival outcomes through CSF1R inhibition in mouse models, highlighting its potential for clinical application. Ongoing clinical trials further investigate this approach, aiming to enhance treatment efficacy for patients with GBM. This review concludes by emphasizing the significance of repolarizing macrophages as a novel therapeutic opportunity in GBM management, alongside emerging trends and future research directions that could lead to breakthroughs in treatment strategies.

## Graphical abstract

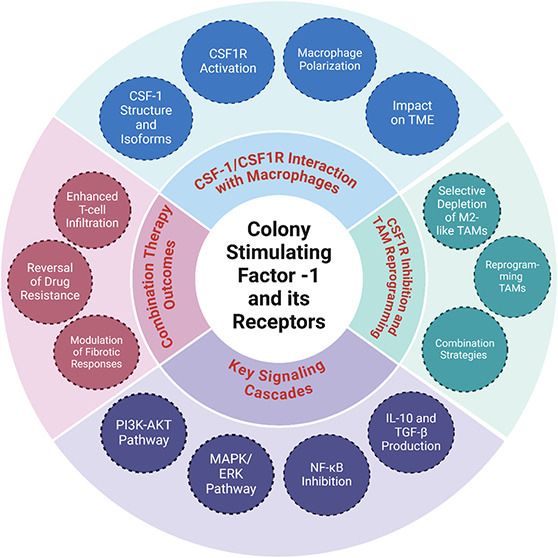

Glioblastoma multiforme (GBM) is a primary brain neoplasm consisting of genetically and phenotypically heterogeneous groups of tumors.^1–3^ With a median overall survival of roughly 15 months, this WHO grade IV brain tumor is among the most severe types of brain cancer.^4,5^ Despite the advances in glioblastoma treatment, which include combinational therapies like surgical removal along with chemotherapy using Temozolomide (TMZ) and radiation therapy, less than one-third of the patients survive for more than two years.^6,7^ Additionally, these patients show a highly variable response to chemotherapy, and the recurrence of the tumor is also frequently observed. A few challenges to current treatment are that GBM is highly infiltrative, has heterogeneity, a blood-brain barrier (BBB) that hinders drug delivery, and an immunosuppressive microenvironment.^8,9^ Although immunotherapeutics have shown promise in other cancers, their efficacy in glioblastoma remains limited due to the aforementioned challenges.

In addition to the cancer cells, the tumor microenvironment (TME) of GBM is a very complex and diverse system that includes different resident brain and immune cells along with cells traveling through the tumor, including immune cells generated from bone marrow.^10,11^ Changes in cellular components, cell-to-cell interaction, and cellular metabolic products, including chemical elements like pH and oxygen levels, have a significant impact on TME.^12^ GBM cells can communicate directly via their TME through cell-surface chemicals or indirectly by apocrine or paracrine signaling through growth factors, cytokines, and extracellular conduits.^13^

Glioma TME contains three different types of components. These include cellular, noncellular, and chemical components.^14,15^ The cellular component involves glioma cells and glioma stem cells, which contribute to the heterogeneity of GBM.^16,17^ They are implicated in tumorigenesis, i.e., they can differentiate into multiple cell types and self-renew, aiding the sustainability of tumors and adaptability to therapeutics.^18^ Different immune cells like microglia, myeloid-derived suppressor cells (MDSCs), tumor-associated macrophages (TAMs), and lymphocytes are also included in cellular components.^19^ Noncellular components comprise extracellular matrix (ECM) and BBB. Modifications in ECM composition can facilitate the infiltration and migration of tumor cells in GBM where BBB is often disturbed in GBM, leading to enhanced permeability that allows for tumor growth while complicating drug delivery.^20,21^ Similarly, chemical components include hypoxia and acidosis.^22^ The presence of hypoxic areas in the tumors aids metabolic adaptations in GBM cells, thus enhancing their survival and promoting aggressive behaviors. Meanwhile, tumor acidosis affects cellular metabolism, leading to the stemness of GBM cells, complicating the treatment process.^23,24^

Macrophages are crucial in TME of GBM, as they significantly implicate tumor progression, immune response, and therapeutic outcomes. TAMs, being abundantly available immune cells in TME, exhibit remarkable plasticity, aiding them to adopt various functional states that can promote or inhibit tumor growth.^25^ They are a versatile immune system component, mainly responsible for maintaining tissue homeostasis and coordinating immune responses.^26^ Phagocytosis, cytokine production, and tissue repair are general functions of macrophages. Phagocytosis is a phenomenon wherein macrophages consume and digest cellular debris, pathogens, and dead cells, leading to tissue repair and homeostasis.^27^ Moreover, they secrete a broad range of cytokines modulating inflammation and employ other immune cells to sites of injury or infection.^28^ Macrophages facilitate wound healing and promote angiogenesis by releasing growth factors.

Concerning cancer, especially GBM, macrophages have a dual task in suppressing or promoting the growth of tumor. Macrophages often polarize into M1 (pro-inflammatory) and M2 (anti-inflammatory) phenotypes, essential for their functions.^29^ M1 macrophages are associated with antitumor activity, having the potential for presenting antigens and resulting in pro-inflammatory cytokines that activate T cells. It is observed that M1 macrophages are limited in GBM due to their immunosuppressive TME.^30^ Conversely, M2 TAMs are frequent in GBM and aid tumor growth.^31^ They secrete anti-inflammatory cytokines that restrain antitumor immune responses and support tumor progression via angiogenesis and ECM remodeling.^32^ The GBM cells-TAMs interaction is important for tumor progression. These interactions lead to the secretions of factors by TAMs that lead to the polarization of macrophages toward pro-tumor phenotypes.^33^

Several pathways are involved in macrophage repolarization, like colony-stimulating factor 1 (CSF-1) and its receptor CSF1R, CD47, CD40, Toll-like receptors (TLRs), and Janus kinase/signal transducers and activators of Transcription (JAK/STATs) pathway. CSF-1 is important in survival, proliferation, and differentiation of macrophages.^34^ Furthermore, it is also essential in maintaining the population of macrophages and contributing to polarization toward a pro-tumor M2 phenotype.^35,36^ CD47 is a protein that gives a “don’t eat me” signal to prevent phagocytosis by TAMs. Blocking of CD47 leads to enhanced phagocytosis, which may facilitate the repolarization of macrophages.^37–40^ Adversely, CD40 is a costimulatory receptor enhancing macrophage activation when occupied. It modifies the immune response by promoting pro-inflammatory cytokine production and interaction with TLRs, influencing macrophage repolarization.^41^ TLRs are essential for recognizing pathogen-associated molecular patterns and initiating innate immune responses. TLRs promote M1 polarization through signaling pathways resulting in the production of inflammatory cytokines, enhancing antitumor immunity.^42–45^ Similarly, the JAK/STAT pathway is crucial for mediating cytokine effects involved in macrophage repolarization.^46^ This review emphasizes the role of CSF-1 and its receptor in macrophage repolarization for its significant influence on macrophage survival, differentiation, and polarization in treating GBM. It is found that CSF-1 signaling has shown a significant influence on the modulation of balance concerning pro-inflammatory M1 and anti-inflammatory M2 phenotypes.

## Significance of CSF-1 and CSF1R

CSF-1, also called macrophage colony-stimulating factor (M-CSF), is an important cytokine in hematopoietic system that modulates macrophage growth, differentiation, and function. It is crucial in various physiological processes involving immune responses, tissue homeostasis, and development. CSF-1 is a glycoprotein that exists in three main isoforms: a glycosylated secreted form, a proteoglycan form, and a membrane-bound form. Various cell types, including fibroblasts, endothelial cells, and bone marrow stromal cells, produce the secreted glycoprotein. The proteoglycan isoform is believed to have enhanced bioactivity due to its resistance to proteolysis, while the membrane-bound form plays roles in cell–cell interactions and localized signaling. These isoforms allow CSF-1 to exert diverse functions in cellular signaling pathways and macrophage biology.^47^ It is also essential for the survival and proliferation of monocytes and macrophages. It promotes the differentiation of hematopoietic stem cells into monocytes and subsequently into macrophages, which are important constituents of the immune system^48^ and influences various macrophage functions, including phagocytosis, cytokine production and migration, thus is crucial in regulating immune responses during inflammation and infection. In addition to its immunological functions, CSF-1 is involved in tissue homeostasis and developmental processes. It influences the function and maintenance of osteoclasts, which are cells responsible for bone resorption and are crucial for normal skeletal development.^49^ Furthermore, CSF-1 has been implicated in wound healing, which benefits in recruitment and activation of macrophages to sites of tissue injury, promoting repair and regeneration processes.^50^

CSF1R is the specific receptor for CSF-1, classified as a receptor tyrosine kinase (RTK).^51^ It is predominantly expressed on the surface of monocytes, macrophages, and dendritic cells, crucial in mediating the biological effects of CSF-1.^52^ The binding of CSF-1 induces dimerization of CSF1R, leading to its autophosphorylation and various intracellular signaling pathways activation, including PI3K-AKT and MAPK. CSF1R signaling is essential for the differentiation of monocytes into macrophages and tissue macrophage maintenance. The CSF1R activation leads to enhanced macrophage survival, proliferation, and activation, which are critical for effective immune responses. Importantly, studies have revealed that the absence of CSF1R has detrimental effects on macrophage populations, leading to impaired immune function and potentially contributing to pathological conditions. Their integral roles in immune regulation, CSF-1, and CSF1R, are significant in various pathological conditions, including cancers, inflammatory diseases, and metabolic disorders. Tumor-associated macrophages often exhibit upregulated CSF1R expression, facilitating tumor growth and metastasis. Therefore, targeting CSF1R has risen as a promising therapeutic approach to impede the pro-tumorigenic activities of these macrophages and enhance antitumor immunity. Furthermore, modulation of CSF-1/CSF1R signaling presents potential therapeutic advantages in treating chronic inflammatory diseases, such as multiple sclerosis and atherosclerosis, where macrophage activity plays a crucial role in disease progression.^53^ Hence, understanding the intricate mechanisms driving CSF-1 and CSF1R interactions will enhance our ability to develop effective therapies for immune-related disorders and cancers.

## Role of CSF-1/CSF1R in Macrophage Biology

Originating from a common precursor in the bone marrow, the cells in the mononuclear phagocyte system travel through the bloodstream before maturing and activating in different organs or derived from the yolk sac, i.e., microglia in the brain, Kupffer cells in the liver, Langerhans cells in skin, alveolar macrophages in lungs, peritoneal macrophages in peritoneum.^54^ The CSF-1 and CSF1R axis meticulously controls the tissue macrophages’ abundance.^55^ The fact that macrophages alter their nature according to their environment, the signals they get, and the needs of surrounding tissue, that is, whether to kill or repair.^56^ Macrophage CSF-1 signaling and CSF1R drive myeloid progenitors to differentiate into distinct populations of macrophages, monocytes, dendritic cells, and bone-resorbing osteoclasts. The CSF-1/CSF1R axis influences mononuclear phagocytes’ ability to proliferate, differentiate, and survive, along with its functions.^57^

## CSF-1 and CSF1R in Macrophage Proliferation, Differentiation and Polarization

Pluripotent hematopoietic stem cells (HSCs) proliferate and differentiate to produce various blood cell types through the process of hematopoiesis. These self-renewing HSCs generate multipotent progenitor precursors (MPPs), which further differentiate into lymphoid-primed multipotent progenitors (LMPPs), also referred to as multilymphoid progenitors (MLPs). From LMPPs, differentiation proceeds toward the formation of common myeloid progenitors (CMPs).^58^ CMPs give rise to granulocyte-monocyte progenitors (GMPs), a process regulated by the transcription factor PU.1, which is critical for the transition from MPPs to CMPs and subsequently to GMPs.^59^ Further lineage specification within GMPs is controlled by a transcriptional module involving interferon regulatory factor 8 (IRF8), also known as interferon consensus sequence binding protein 1 (ICSBP1). IRF8 binds to the interferon-stimulated response element (ISRE) and regulates the expression of genes responsive to type I interferons (IFN-α/β), directing GMPs to differentiate into either granulocyte progenitors (GPs) or monocyte progenitors (MPs). MPs subsequently differentiate into macrophages, as depicted in Figure 1.^56,60^ CSF1R plays a critical role in the development of both classical and nonclassical monocyte subsets. Its ligand, CSF-1 (also known as M-CSF), is a key cytokine involved in macrophage production. CSF1R is expressed in monocytes, macrophages, and mononuclear phagocyte precursors.^55^ The binding of CSF-1 to CSF1R triggers a cascade of intracellular signaling events, including autophosphorylation of CSF1R and activation of the PI3K-AKT and AMPK pathways, thereby promoting the generation and maturation of macrophages.

**Figure 1:**
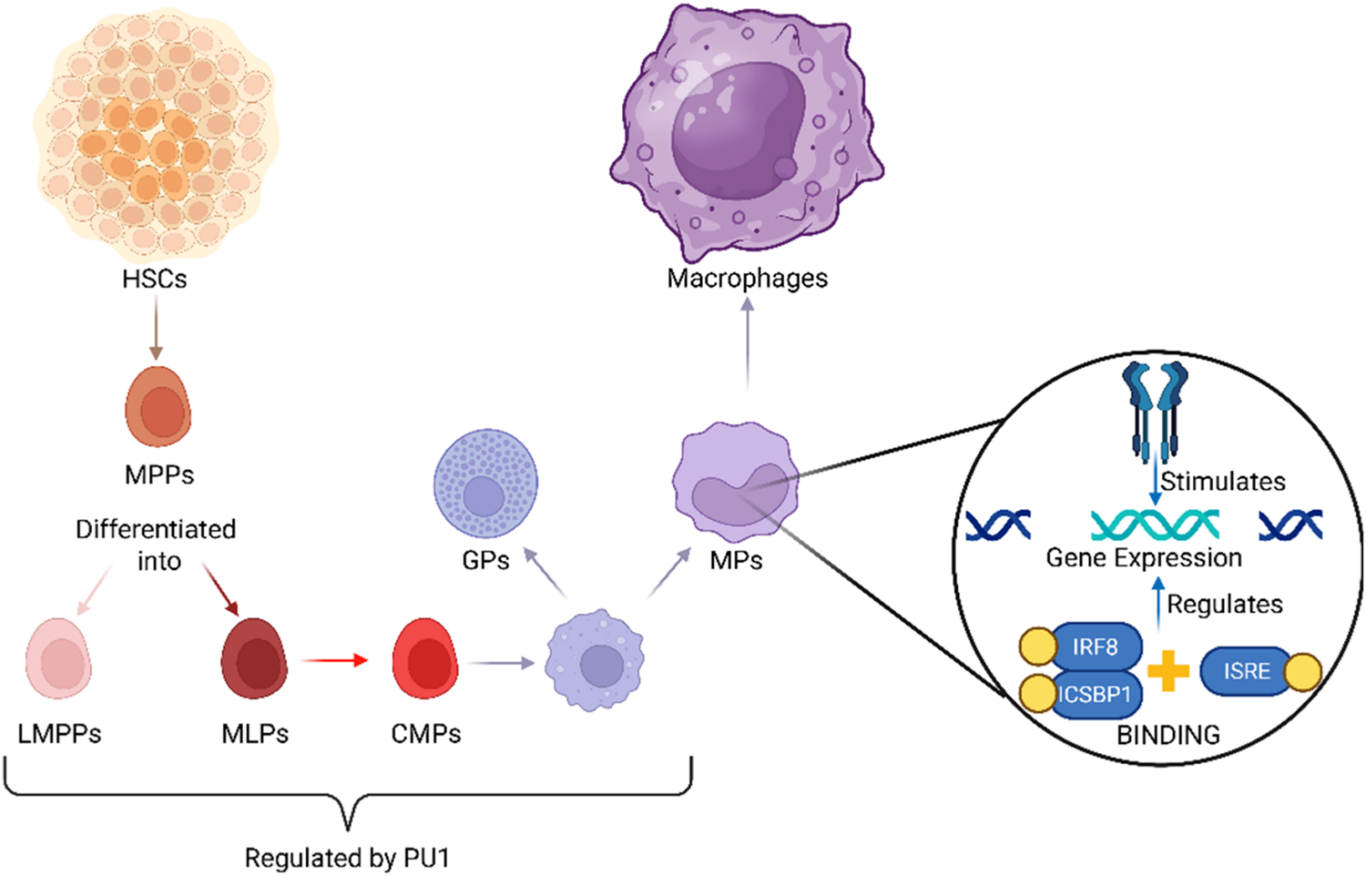
Overview of Macrophage Generation: From HSCs to Diverse Macrophages. *Created in BioRender. Dhas, N. (2025)* https://BioRender.com/61r7mqo.

There are four different types of CSFs: multipotential colony-stimulating factor, commonly known as IL-3 (interleukin-3), granulocyte-macrophage colony-stimulating factor (GM-CSF), granulocyte colony-stimulating factor (G-CSF), and macrophage colony-stimulating factor (M-CSF). The CSF-1 gene encodes CSF-1, which is secreted by osteoblasts, astrocytes, stromal cells, fibroblasts, keratinocytes, endothelial cells, bone marrow, myoblasts, and mesothelial cells.^55^ CSF-1 binds to CSF1R, which has three domains: an intracellular domain with kinase activity, a transmembrane domain, and an extracellular domain that attaches to ligands (CSF-1, IL-34). The binding of CSF-1 to CSF1R employs PI3K and Src to the receptor, activating downstream MAPK and Akt signaling pathways.^61^ Autophosphorylation of CSF1R on different tyrosine residues (such as Tyr-807, Tyr-921, Tyr-974, Tyr-706, Tyr-721, Tyr-559, Tyr-544 and Tyr- 697) initiated after binding of CSF-1.^62^ Different signaling molecules attached to these docking sites are created by phosphorylation, activating distinct signaling pathways.^63^ Tyr-559 phosphorylation stimulates MAPK, extracellular signal-regulated kinase (ERK), and Src family kinases.^64^ PI3K is activated when CSF1R is phosphorylated by Tyr- 721. MAPK, ERK1, and ERK2 are activated by Tyr- 807 phosphorylation, while other signaling pathways are activated by employed Grb2 by phosphorylated Tyr-921/Tyr-697.^65^ The only RTK that is controlled by both interleukin-34 (IL-34) and CSF-1 is CSF1R ligands attached to the extracellular domain’s single-pass transmembrane helix, a linker region, and immunoglobulin (Ig)- like domains­(D1-D5). D1-D3 carries out ligand recognition, and D4-D5 carries out ligand–receptor complex stabilization.^66^ CSF-1 and CSF1R ligand interaction induce CSF1R dimerization and phosphorylation of tyrosine residues, which promotes differentiation, as shown in Figure 2.

**Figure 2:**
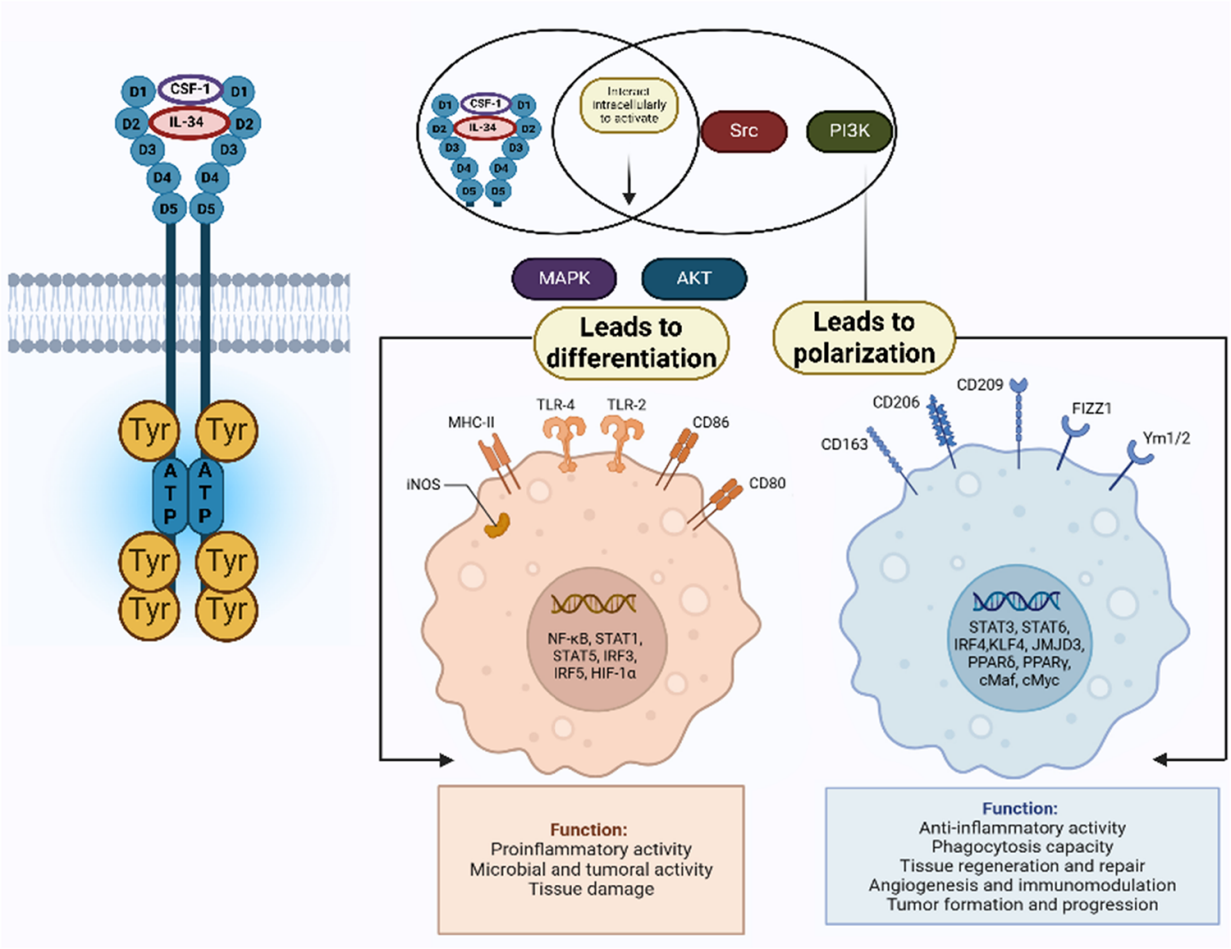
Differentiation of M1 and M2 Macrophage Subtypes Mediated by CSF-1. *Created in BioRender. Dhas, N. (2025)* https://BioRender.com/9h1o30h.

The PI3K signaling pathway triggered by CSF1R is proven to control macrophages’ M1/M2 polarization axis.^64^ AKT1 and m-TORC2 are activated when CSF-1 attaches to CSF1R, initiating a signaling cascade of class I PI3K.^55^ Additionally, the NF-kB (nuclear factor kappa B) mediated M1 signals are inhibited by the activated AKTs. According to literature reports, the signaling triggered by Tyr-721 phosphorylation upregulates the production of genes linked to M2 polarization profile (Arginase (Arg 1) and IL-10) and downregulates pro-inflammatory genes (IL-1β, IL-12, TNF-α, etc.) associated with the M1 macrophage profile.^67,68^ The CSF-1-dependent Tyr-721 and PI-3K pathway ultimately produce mi-RNA (mi-R21), which has been shown to regulate macrophage activation. The mi-R21 also reduces recruitment of Ly6Chigh inflammatory monocytes to the peritoneal cavity, suggesting a conclusive function for the CSF-1-dependent Tyr-721 and PI-3K induced mi-R21 in the macrophage polarization axis.^69^

## Signaling Pathway (Activation by AKT/PI-3K)

Macrophages initiate immunological responses when infections and other damaging stimuli, such as necrotic or apoptotic cell debris activate them.^70^ The extensive terms M1 and M2 phenotype refer to various activation phenotypes that macrophages acquire based on the signals. The PI3K/AKT/mTOR pathway mediates the signals from insulin receptors, cytokine receptors, adipokine receptors, and pathogen-associated molecular pattern receptors.^71^ The AKT pathway incorporates metabolic and inflammatory signals to control macrophage responses and modify their activation profile. Signaling cascades downstream of TLR and cytokine receptors mediate cytokine activation. Proinflammatory cytokines and chemokines are produced due to transcriptional and epigenetic modifications upon activation. The PI3K/AKT pathway and its downstream targets have become key modulators of macrophage activation phenotype.^68,72^ In addition to controlling macrophage migration, survival, and proliferation, the PI3K/AKT pathway coordinates the macrophages’ response to many inflammatory and metabolic stimuli. TLR4 and pathogen recognition receptors, cytokine and chemokine receptors, all modulate downstream signals that regulate the synthesis of cytokines by activating the PI3K/AKT pathways. To produce phosphatidylinositol 3,4,5-triphosphate (PIP3), activated PI-3K type I phosphorylates phosphatidylinositol 4,5-biphosphate (PIP2). AKT and m-TORC2 are further recruited by this PIP3, which also makes it easier for m-TORC2 to activate AKT.^68,73^ Tuberous sclerosis complex (TSC) 1/2 is phosphorylated and rendered inactive by activated (phosphorylated) AKT. Through the regulation of the Ras homologue abundant in the brain (known as Rheb), TSC1/2 inhibition by AKT results in m-TORC1 activation.^73,74^ Limiting proinflammatory responses and amplifying anti-inflammatory responses in TLR-stimulated macrophages depends on the activation of the PI3K/AKT pathway, which has been considered a negative regulator of TLR and NF-kB signaling in macrophages.^75,76^ While activation or overexpression of PI3K or AKT kinases reduced macrophage activation by LPS, TLR-activated nonspecific chemical inhibition of PI3K signaling increased NF-kB activation and inducible NO synthase synthesis, boosting M1-type macrophage responses.^77^ In response to IL-4 or surfactant protein A, PI3K activation has been illustrated as a necessary step in the M2 activation of macrophages.^78,79^

The autophosphorylation and dimerization of many tyrosine residues activate the autoinhibited CSF1R receptors, initiating a signaling cascade that results in the internalization of receptors. The PI3K-AKT and AMPK pathways, which have significance in macrophage proliferation, are part of the cascade, which is started by binding the endogenous CSF-1 and interleukin-34 (IL-34). Both cytokines aid in macrophage survival, growth, and multiplication.^80^

## Role of CSF1R and Macrophages in the Immune System, Tissue Repair, and Healing

The tissue system coordinates inflammation, debris removal, and regeneration, all of which are essential for tissue repair. Upon injury, DAMPs (damage-associated molecular patterns) and PAMPs (pathogen-associated molecular patterns) activate pattern recognition receptors (PRRs) on immune and local cells (e.g., fibroblast, endothelial cells), triggering pro-inflammatory signaling and recruiting innate immune cells, especially monocytes.^81,82^ These monocytes, via CCR2 (chemokine receptor 2) dependent mechanism, migrate to the injury site, where they release pro-inflammatory cytokines and chemokines, amplifying the immune response.^83^ Monocytes develop into macrophages at the site of injury, and when CSF1 attaches to it, the macrophages dynamically change from the M1 to the M2 phenotype by activating CSF1R. M1 macrophages dominate the acute phase, producing cytokines (IL-6, TNF- α), reactive oxygen species, and antimicrobial peptides while clearing debris and presenting antigens. As inflammation resolves, M2 macrophages become prevalent, secreting anti-inflammatory cytokines like IL-10 and growth factors such as TGF-β and PDGF, which promote angiogenesis, ECM remodeling, and stem cell recruitment.^84^ M2 macrophages also interact with adaptive immune cells (e.g., T helper 2 and regulatory T cells) to resolve inflammation. Subtypes of M2 macrophages, such as M2a (regenerative), M2b and M2c (anti-inflammatory), reflect functional diversity. M2a macrophages, driven by IL-4, are particularly associated with fibrosis and regeneration, while others, like M2d, promote angiogenesis via VEGF secretion. Although macrophages exhibit a spectrum of activation states, the M1/M2 paradigm remains a useful framework to describe their functions in tissue repair.^85^

## Macrophages in the Glioblastoma Microenvironment

Macrophages, especially TAMs, are significant in GBM. This role is multifaceted and important to understand tumor progression and resistance therapy. Overexpression of CSF1R usually occurs in TME in various types of cancer. This overexpression led to TAM uptake in cancerous areas.^86^ Increase the level of CSF-1 associated with cancer cell proliferation, migration, drug resistance, and stemness. The overexpression of CSF1R in GBM can significantly affect tumor progression through its impact on cell proliferation and migration. Higher CSF1R levels in glioma cells are associated with greater viability through increased expression of *K*_i_-67 and more significant colony formation ability. Additionally, CSF1R suppression prevents entry into the S phase by causing p27 production, which impairs cell cycle progression. Furthermore, CSF1R overexpression can lead to migration by altering the activity of ERK1/2 signaling. These findings highlight the impacts of overexpression of CSF1R in glioblastoma progression and poor survival rate.^87^

## TAM Phenotype and Tumor Progression

M1 and M2 are two distinct phenotypes of TAMs that coexist in the TME. Because of their plasticity, TAMs can change between phenotypes in response to environmental signals.^88^ Researchers have found a variety of markers to differentiate TAMs, even though there is not a single signature that is unique to them. M2 macrophages express markers including CD23, CD163, CD206, CD1a, CD1b, CD93, and CD226, while M1 macrophages are identified by markers like CD86, CD64, CD16, CD120b, TLR2, and SLAMF7.^89^ M1 macrophages aid in the removal of damaged tissues by attracting neutrophils and enhancing the inflammatory response. The improved phagocytic capabilities of M1 macrophages contribute to the removal of debris, disinfection, and removal of bacteria, and clearance of wasted neutrophils.^90^

Pro-inflammatory cytokines such as IL-12 and TNF-α are secreted by M1 TAMs, which also stimulate CD8+ T cells and NK cells, activate Th1 immune response, and have antitumor effects in early stages of tumor development (nascent tumors). Conversely, M2 TAMs assist tumor progression in later stages (developed tumors) by secreting immunosuppressive factors, including PD-L1, TGF-β, and IL-10; they also encourage angiogenesis through growth factors such as vascular endothelial growth factor (VEGF) and facilitate tumor invasion and metastasis through the release of molecules like IL-6 and TGF-β.^91^

TAMs promote tumor growth, angiogenesis, invasion, and immune evasion, all of which are critical processes in GBM progression. In TME, TAMs frequently take on an M2-like phenotype, identified by CD204 and CD163, associated with low survival and aggressive tumor growth. These TAMs release growth factors and cytokines like VEGF, TGF-β, and IL-10 that promote development, stemness, and resistance to treatment of glioblastoma cells. TAMs also stimulate angiogenesis by generating VEGF, IL-6, and IGFBP1, which help the tumor grow new blood vessels to supply oxygen and nutrients, especially when oxygen levels are low.^92,93^ Furthermore, they promote glioblastoma cell invasion and metastasis by altering ECM with the help of substances like MMPs and CCL8.^94^

## Feedback Loops and TAMs

Activation of TAMs by CSF-1 leads to the secretion of various factors that promote tumor growth and invasion. TAMs secrete various bioactive molecules like IL-10, VEGF, and EGF, which significantly influence tumor cells. These factors induce processes like epithelial-mesenchymal transition, leading to the acquisition of migratory and invasive properties in tumor cells.^91^ Positive feedback loops are caused by the interaction of TAMs and tumor cells in GBM and other cancers. These feedback loops significantly affect tumor progression, evasion, and resistance to treatment. There are various positive feedback loops in GBM like NFAT1-C3a-C3aR Feedback Loop,^95^ UPF1/circRPPH1/ATF3,^96^ CircRNF10/ZBTB48/IGF2BP3,^97^ NTS/NTSR1 and Wnt/β-Catenin Signaling Feedback Loop and c-myc/XTP6/NF-κB^98,99^ Feedback Loop are explained in Table 1.

**Table 1:** Feedback Loops in GBM

positive feedback loop	explanation	refs
NFAT1-C3a-C3aR Feedback Loop	In GBM, NFAT1 increases C3 transcription, leading to C3a secretion and M2-like polarization of TAMs. This activates the Ca2+-NFAT1 pathway, forming a positive feedback loop. Blocking this loop with a C3aR inhibitor reduces glioma growth.	^95^
UPF1/circRPPH1/ATF3	UPF1 binds to circRPPH1, allowing it to interact with ATF3, promoting UPF1 and Nestin transcription. This feedback loop boosts Nestin expression and tumor growth in glioma stem cells (GSCs). Targeting circRPPH1 decreases GSC proliferation.	^100^
CircRNF10/ZBTB48/IGF2BP3	Interaction between CircRNF10 and ZBTB48 promotes HSPB1 and IGF2BP3 protein expression, which aids iron metabolism and ferroptosis defense.	^97^
NTS/NTSR1 and Wnt/β-Catenin	The neurotensin (NTS) pathway, via NTSR1, activates Wnt/β-catenin, essential for cell growth. NTSR1 strengthens Wnt protein expression, enhancing NTSR1 expression, creating a feedback loop that boosts GBM growth.	^98^

## Targeting CSF1R as a Therapeutic Approach for Glioblastoma

Targeting the CSF1R presents a promising therapeutic strategy for GBM by influencing TAMs. CSF1R is substantial for the survival and differentiation of macrophages, particularly those that promote tumor growth.^101^ CSF1R inhibitors reprogram TAMs to an M1-like state, enhancing antitumor immunity despite reducing monocyte recruitment. Combination therapies (e.g., CD40 agonists) may address monocyte-dependent limitations. The shift of M2 to M1 diminishes their tumor-supporting roles while potentially preserving their antitumor functions.^102^ This reprogramming is facilitated by glioma-secreted factors such as GM-CSF and IFN-γ, which help maintain TAM survival even when CSF1R is blocked.^103^ Additionally, by enhancing T-cell infiltration and overall therapy results, CSF1R inhibitors act more effectively when combined with other medications, such as immune checkpoint inhibitors like PD-1 antibodies.^104^

## Differential Macrophage Activation

Differential repolarization denotes the process by which macrophages can change their function, mainly between M1-type and M2-type, as both play a crucial role in their functional ability: M1-type macrophages enhance inflammation, while M2-type macrophages inhibit it. Macrophages are primary resident cells of the TMEs that can support growth and enable tumor evasion mechanisms, so this switching ability can be particularly significant. Hence, understanding the metabolic pathways and signaling processes involved in this repolarization is essential for developing effective treatments that target these macrophages.^105^ **M1 Macrophages** are activated by interferon-γ (IFN-γ) and lipopolysaccharides (LPS). They produce inflammatory substances that are crucial in combating infections and tumors. **M2 Macrophages** are triggered by cytokines like interleukin-4 (IL-4) and interleukin-13 (IL-13). M2 macrophages facilitate tissue repair and decrease inflammation.^106,107^ However, this anti-inflammatory response can inadvertently promote tumor growth. A promising therapeutic approach in cancer treatment is to repolarize M2 macrophages, often found in tumors, into M1 macrophages so they can effectively tackle cancer.^108^ This repolarization can be achieved by targeting specific metabolic pathways active in M2 macrophages or using certain cytokines that encourage M1 polarization.^109^ The differences between M1 and M2 macrophages from the perspective of GBM are evident in their activation, metabolism, cytokine production, and functions.^26^ M1 macrophages, activated by signals such as IFN-γ, contribute to tumor suppression, while M2 macrophages, activated by IL-4, support tumor growth.^110^

M1 macrophages primarily rely on aerobic glycolysis for energy and produce pro-inflammatory cytokines, enhancing immune responses.^111^ Contrarily, M2 macrophages exhibit oxidative phosphorylation and release cytokines that reduce inflammation, which can promote the growth of tumors. Repolarization techniques try to change M2 macrophages into M1-like phenotypes to increase the efficacy of GBM therapy.^112^ To target tumor cells, macrophage function in glioblastoma must change from a pro-tumorigenic M2 phenotype to a tumor-suppressing M1 phenotype.^56^ M1 macrophages produce pro-inflammatory cytokines and nitric oxide, whereas M2 polarization promotes tumor growth and immune suppression.^111^ Blockage of pathways like PI3K-γ can encourage the polarization of M1, increase the effectiveness of the treatments in GBM, and counteract TMZ resistance.^113^ Thus, macrophage polarization plays a vital role in the TME.

## Role of GM-CSF and IFN-γ in Anticancer Macrophage Activation

IFN-γ and GM-CSF are crucial for activating antitumor macrophages, each fulfilling distinct and essential roles in the immune response against neoplasms.^114^ GM-CSF is crucial in facilitating the proliferation, maturation, and activation of numerous immune cells, such as macrophages, dendritic cells, and neutrophils. This cytokine, therefore, accelerates the overall capability of the immune system to combat tumors by migrating the immune cells to tumor sites, improving subsequent antigen presentation and T-cell activation. However, GM-CSF also exhibits a dualistic effect. Simultaneously, it bolsters antitumor immune responses, and it can inadvertently support tumor growth by upregulating the expression of PD-L1 on tumor cells, which aids in immune evasion.^115^

Conversely, IFN-γ acts as a pro-inflammatory cytokine that activates macrophages to adopt the M1 phenotype, thus showing a highly intensified response against cancer cells.^76^ The activation causes increased production of inflammatory molecules that increase the potential of the lysis of cancerous cells by macrophages owing to increased synthesis of nitric oxide and improved antigen presentation.^116^ When deployed synergistically, GM-CSF and IFN-γ significantly enhance macrophage activation: GM-CSF recruits and differentiates immune cells, while IFN-γ amplifies their functionality against tumors. Collectively, these cytokines generate a formidable immune response against malignant cells.^117^ Nevertheless, it is imperative to meticulously regulate their levels in therapeutic contexts to mitigate the risks associated with excessive GM-CSF activity, which could lead to enhanced tumor proliferation.^118^ Understanding the interaction between GM-CSF and IFN-γ within the TME is critical to developing immunotherapeutic approaches, particularly for aggressive malignancies such as GBM. Precise modulation of these cytokines in oncological therapies can enhance tumoricidal effects while diminishing adverse effects.

## Therapeutic Inhibition of CSF1R

Inhibiting the CSF1R represents a novel therapeutic approach to reprogramming TAMs within the TME in GBM. Since CSF1R is essential for monocyte and macrophage survival and proliferation, inhibiting this receptor might significantly reduce the type of pro-tumor M2-like TAMs while either preserving or enhancing the activity of anticancer M1-like macrophages depicted in Figure 3.^119^ The figure illustrates the different immune environments that regulate macrophage polarization and its function in the TME. Under immunosuppressive conditions, various factors like CSF-1, IL-4, IL-10, IL-13, and hypoxia drive the differentiation of monocytes into M2-like TAMs, which are CSF1R-dependent. These TAMs, with other immune cells like dendritic cells (DCs), regulatory T cells (Tregs), and Type 2 helper cells, release immunosuppressive factors facilitating tumor progression. Inhibition of CSF-1/CSF1R primarily targets this pathway by blocking the survival and proliferation and utilizing M2-like TAMs responsible for immuno-suppression and tumor growth. This block helps in shifting the balance toward an immune response that fights the tumor, which is similar to the immuno-stimulation, which promotes M1-like macrophages and antitumor activity.^120^ Therapeutic inhibition of CSF1R has arisen as a promising approach to improve antitumor immunity. Evaluations of the use of CSF1R inhibitors like BLZ945 and PLX3397 demonstrated that evident regression of established tumors and improved survival were achievable in preclinical models of GBM. Interestingly, rather than depleting TAMs, CSF1R inhibition appears to ″re-educate″ them within the TME, decreasing markers associated with M2 polarization and impairing their tumor-promoting functions while enhancing their ability to present antigens and producing pro-inflammatory cytokines.^121^ Moreover, combining CSF1R inhibitors with other therapies, such as radiotherapy (RT), has shown improved efficacy by mitigating the increase in M2 polarized TAMs that often occur following radiation treatment. This combination not only enhances the antitumor effects of RT but also restores the sensitivity of glioma cells to other therapies by altering the TME, making it less conducive to tumor growth and resistance.^122^ Targeting CSF1R represents a multifaceted approach that affects macrophage behavior and enhances the effectiveness of existing GBM therapies.

**Figure 3:**
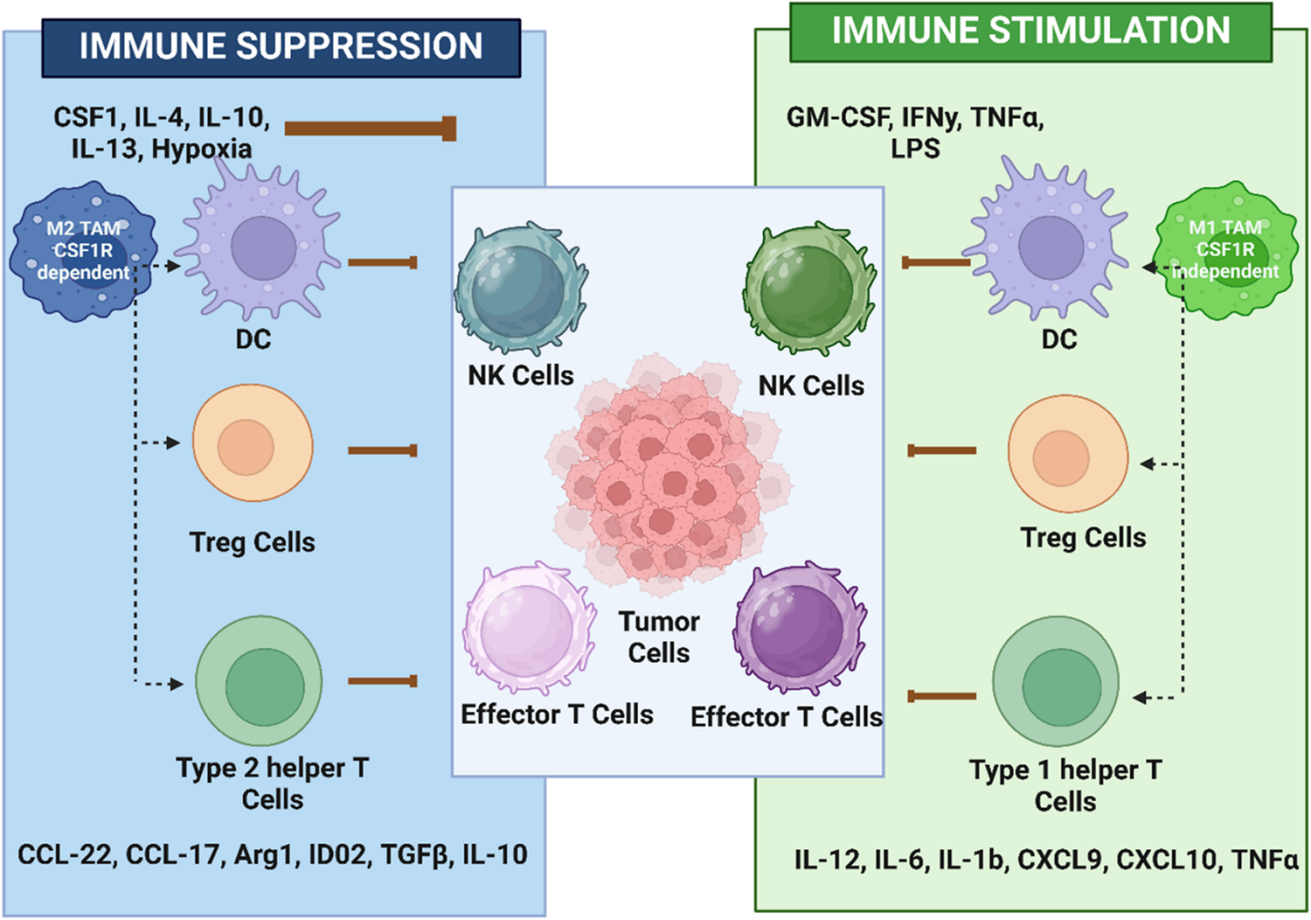
Regulation of TAMs and their effect on the tumor microenvironment. Adapted with permission from.^120^ Copyright 2017 μ_B_J Publishing Group Ltd. and Society for Immunotherapy of Cancer Michael A. Cannarile.

## Mechanisms of CSF1R Inhibition

CSF1R inhibition involves strategies designed to block the CSF1R, which plays a vital role in the survival and function of macrophages.^53^ These could be accomplished using small molecule inhibitors or monoclonal antibodies, which reduce TAM’s recruitment and survival within the TME. The inhibition of CSF1R depletes M2-like macrophages, best known for their role in promoting tumor growth, but also increases the population of M1-like macrophages that can mediate antitumor responses. Due to this transition from pro-tumor to antitumor activity, the levels of immunosuppressive cytokines like IL-10 and TGF-β decreased. In contrast, pro-inflammatory cytokines such as TNF-α and IL-12 significantly increased.^123,124^ Additionally, CSF1R inhibition may impact other immune cells, creating an environment that bolsters immune responses against tumors. Furthermore, combining CSF1R inhibitors with other therapies, such as radiation, can enhance overall effectiveness by preventing the activation of TAMs, which often contributes to resistance against treatments.^120^ In summary, targeting CSF1R diminishes dysfunctional macrophages in glioblastoma and modifies the immune response, potentially leading to improved patient outcomes.

The study by Quail et al. demonstrated that blocking CSF1R in glioma models significantly remodels the TME by reprogramming TAMs toward a less immunosuppressive phenotype. This intervention was shown to prolong survival and impede glioma progression. Specifically, the study employed a CSF1R inhibitor (PLX3397) in both genetically engineered mouse models and orthotopic glioma models, revealing substantial therapeutic benefits. Notably, treatment with PLX3397 extended median survival from 27 to 40 days (*p* < 0.0001), indicating a statistically significant improvement. Histological analysis showed reduced tumor cell proliferation and increased macrophage infiltration, with a shift toward a pro-inflammatory phenotype. Additionally, this therapy resulted in the downregulation of immune-suppressive genes such as Arg1 and Il10, while enhancing the expression of major histocompatibility complex (MHC) class II and Il12, indicating immune activation. Interestingly, tumors eventually developed resistance to CSF1R inhibition without a reduction in tumor-associated macrophage (TAM) infiltration, indicating an adaptation within the tumor-immune landscape. A combination therapy that included IGF-1R blockade restored sensitivity to treatment and further prolonged survival. This suggests that there is a compensatory mechanism involving insulin-like growth factor pathways. Collectively, these results demonstrate that therapeutic inhibition of CSF1R effectively alters the immunological environment of gliomas and provides a statistically significant survival benefit, highlighting the potential for combinatorial strategies in glioblastoma treatment.^119^

Similarly, Tanaka and colleagues investigated the therapeutic inhibition of Colony-Stimulating Factor 1 Receptor (CSF1R) as a promising strategy to suppress TAMs and enhance antitumor immunity, particularly in glioma models. Using a highly selective small molecule inhibitor, referred to as PLX3397, the study demonstrated a significant depletion of TAMs, resulting in improved immune responses and reduced tumor burden. Statistically, treatment with PLX3397 alone led to about a 70% reduction in TAMs in glioma-bearing mice, accompanied by an increased infiltration of cytotoxic T lymphocytes. Furthermore, when PLX3397 was combined with radiotherapy, the effects were synergistic, leading to prolonged survival in GL261 glioma models, with the median survival extending from 24 days in the control group to 43 days in the combination group. Histological analysis revealed a marked decrease in immunosuppressive macrophages and increased levels of pro-inflammatory cytokines, indicating a reprogramming of the tumor microenvironment. These findings support targeted inhibition of CSF1R as an effective therapeutic approach to re-educate the tumor immune landscape and improve clinical outcomes, especially when integrated with other modalities such as radiation. This study highlights the potential utility of CSF1R inhibitors like PLX3397 as part of a combined immunotherapeutic strategy for aggressive brain tumors like glioblastoma.^125^

Study conducted by Chipman et al. explores the role of tumor-associated macrophages (TAMs) in tumor progression using genetically engineered mouse models (GEMMs) of breast and lung cancer. The authors specifically assess whether therapeutic inhibition of the colony-stimulating factor 1 receptor (CSF1R), a key regulator of macrophage survival and recruitment, impacts tumor development. Using the CSF1R inhibitor BLZ945, they achieved effective TAM depletion in both breast and lung tumor models. Surprisingly, despite significant reduction in macrophage populations (confirmed by immunohistochemistry and flow cytometry), there was no significant impact on tumor burden, progression, or immune cell composition. Tumor sizes, histological grading, and survival outcomes remained comparable between treated and untreated groups. Quantitative analyses showed that while CSF1R inhibition drastically reduced F4/80^+^ and IBA1^+^ macrophages, it did not alter the number or proliferation of epithelial tumor cells. These findings highlight that, in certain cell lineage-based tumor contexts, TAMs may not be essential for tumor progression, and therapeutic targeting of CSF1R may have limited efficacy, challenging the generalization of TAM-targeted strategies in cancer immunotherapy.^126^

## Selective Depletion of Tumor-Promoting TAMs

Selective depletion of tumor-promoting TAMs is a critical strategy for enhancing antitumor immunity in GBM. CSF1R inhibition selectively reduces M2-like TAMs associated with immune suppression and tumor progression while preserving or enhancing M1-like macrophages that exhibit antitumor properties.^127^ This selective targeting is achieved using agents such as PLX3397 and BLZ945, effectively decreasing the density of M2-like TAMs within the TME without compromising overall immune function. Importantly, this selective targeting helps preserve or enhance the activity of M1-like macrophages, vital for initiating and sustaining antitumor immune responses by producing pro-inflammatory cytokines like TNF-α and IL-12.^128^

Furthermore, studies have shown that CSF1R inhibition can increase the infiltration of CD8+ T cells into tumors, enhancing the efficacy of therapies such as anti-PD-1 by facilitating T cell migration and reprogramming the immunosuppressive environment.^129^ Combining CSF1R inhibitors with other treatments, including radiotherapy or immune checkpoint inhibitors, has demonstrated synergistic effects in reducing tumor growth and improving overall survival rates in preclinical models.^120^ This multifaceted approach alters the balance between pro-tumor and antitumor immune cells. It reshapes the TME to create an environment conducive to effective antitumor responses without compromising overall immune function.^130^ Overall, selective depletion of pro-tumor TAMs through CSF1R inhibition represents a strategic method to enhance antitumor immunity in GBM and other cancers while minimizing adverse effects associated with broad-spectrum immunosuppression.

## Sparing of Antitumor Macrophages

Sparing antitumor macrophages while selectively depleting tumor-promoting TAMs is crucial in cancer immunotherapy for GBM. CSF-1R inhibition achieves this balance by reducing M2-like TAM populations without significantly affecting M1-like macrophages. Mechanisms include blocking immunosuppressive signals produced by TAMs, such as IL-10 and TGF-β, which restores M1-like macrophage functionality and enhances their ability to activate T cells against tumors.^131^ Research has shown that reprogramming TAMs through exposure to specific stimuli, such as iron or cytokines like IFN-γ, can enhance their antitumor activities; for instance, iron-loaded TAMs have been identified as pro-inflammatory and capable of directly killing tumor cells, suggesting that delivering iron to the TME could serve as an adjuvant therapy to promote anticancer immune responses.^132^

Additionally, strategies that block immunosuppressive signals produced by TAMs, for example, IL-10 and TGF-β, can help restore the functionality of M1-like macrophages and enhance their ability to activate T cells and other immune components against tumors. Moreover, combining CSF1R inhibitors with other therapies, such as chemotherapy or checkpoint inhibitors, has demonstrated synergistic effects in improving therapeutic outcomes. By focusing on the spatial distribution and functional states of different macrophage populations within the TME, researchers aim to develop more effective treatment regimens that target tumor-promoting TAMs and bolster the antitumor activities of beneficial macrophage subsets.^133^ These strategies highlight the potential for manipulating macrophage dynamics in the TME to improve cancer therapy outcomes while minimizing adverse effects associated with broader immune suppression.

## Preclinical Evidence and Models

Preclinical research is essential for bridging laboratory discoveries and clinical applications, involving rigorous testing of potential therapies in models that assess their safety and efficacy. This section will highlight key findings from these models, focusing on the promising results associated with CSF1R inhibition, which has shown improved survival outcomes in GBM (Table 2). Understanding these preclinical findings is crucial for translating them into effective clinical therapies.

**Table 2:** Overview of Pathways, *in Vivo* Models, and Outcomes of the Preclinical Studies

study	explored pathway	*in vivo* models	outcomes	refs
DBLZ-Conjugate for CSF1R Inhibitor Delivery in GBM Models by Liaw et al.	The pathway involved in the inhibition of CSF1R on TAMs using DBLZ is explored in this study.	Mouse models bearing brain tumors	Single systemic dose enhanced life quality, survival, T cell infiltration, immune activation, and drug concentration at the tumor site.	^134^
Preclinical Research on CSF1R Inhibition Effects on Glioma Models by Watson et al.	Pathways explored in this study are related to wound inflammation, EMT, and ECM production post-CSF1R inhibition.	PDG transgenic mouse model.	BLZ945 treatment resulted in tumor regression, increased survival, and antitumor efficacy.	^135^
M-CSF and GM-CSF Signaling Functions in Differentiation and Polarization of TAMs in Tumor Models in Several Models by Van Overmeire et al.	M-CSFR signaling, especially its role in recruitment, extravasation, proliferation, and maturation of tumor-infiltrating Ly6Chi monocytes is explored.	C57BL/6 mice, ubiquitin-GFP mice, and genetically modified mice.	M-CSFR signaling blockade impairs extravasation of tumor-infiltrating Ly6Chi monocytes, affecting their differentiation and influencing tumor growth and response to therapies.	^136^
Anti-CSF1R antibody AFS98 inhibits bone-marrow-derived macrophages and affects glioma-bearing mice’s response by Stessin et al.	mTOR pathway and macrophage polarization to inhibit recurrent GBM.	Orthotopic xenograft model and surgical resection model.	Targeting the mTOR pathway and repolarizing macrophages effectively inhibits GBM growth and recurrence, supporting neurological recovery postsurgery.	^137^
CSF1R inhibitors are a key target for the treatment of glioblastoma and other neuroinflammatory illnesses by van der Wildt et al.	CSF1R expression in glioblastoma macrophages and microglia.	Mouse model of acute and chronic neuroinflammation.	High CSF1R uptake in the brain, specific binding to CSF1R, decreased brain uptake with BLZ inhibitor; metabolic stability, not a substrate for BBB efflux transporters.	^140^

A study by Liaw et al. aimed to assess the effectiveness of a new dendrimer conjugate for the targeted delivery of CSF1R inhibitors BLZ945 to TAMs in orthotopic GBM models. The results of this study suggested that dendrimer-conjugated BLZ945 significantly improved TAM reprogramming, promoted cytotoxic T-cell infiltration, prolonged survival, and enhanced motor and behavioral markers of disease progression evaluated after 10-day post-tumor inoculation and analysis using confocal microscopy. The TAMs in untreated mice showed an ameboid morphology, which indicated immunosuppressive activation. However, DBLZ-treated mice exhibited a 2-fold increase in surface area to volume ratio than controls, signifying partial recovery of TAMS compared to controls. Free BLZ treatment did not result in significant morphological changes in TAMs compared to controls. Moreover, DBLZ treatment significantly reduced Arg-1 expression in TAMs compared to control and free BNLZ. This observation indicates the shift from immunosuppressive activation. A substantial enhancement in CD8+ cytotoxic T-cell infiltration in tumors treated with DBLZ was noted with an average of 127.9 ± 17.4 cells per 0.1 mm^2^, indicating approximately a 50% increase depicted in Figure 4. This targeted immunotherapeutic approach showed a potential outcome with lower doses than previous research, suggesting its potential for clinical translations in improving patient outcomes via localized alteration of tumor immune response. It was concluded that the novel approach could be a valuable strategy for improving prognosis in GBM patients.^134^

**Figure 4:**
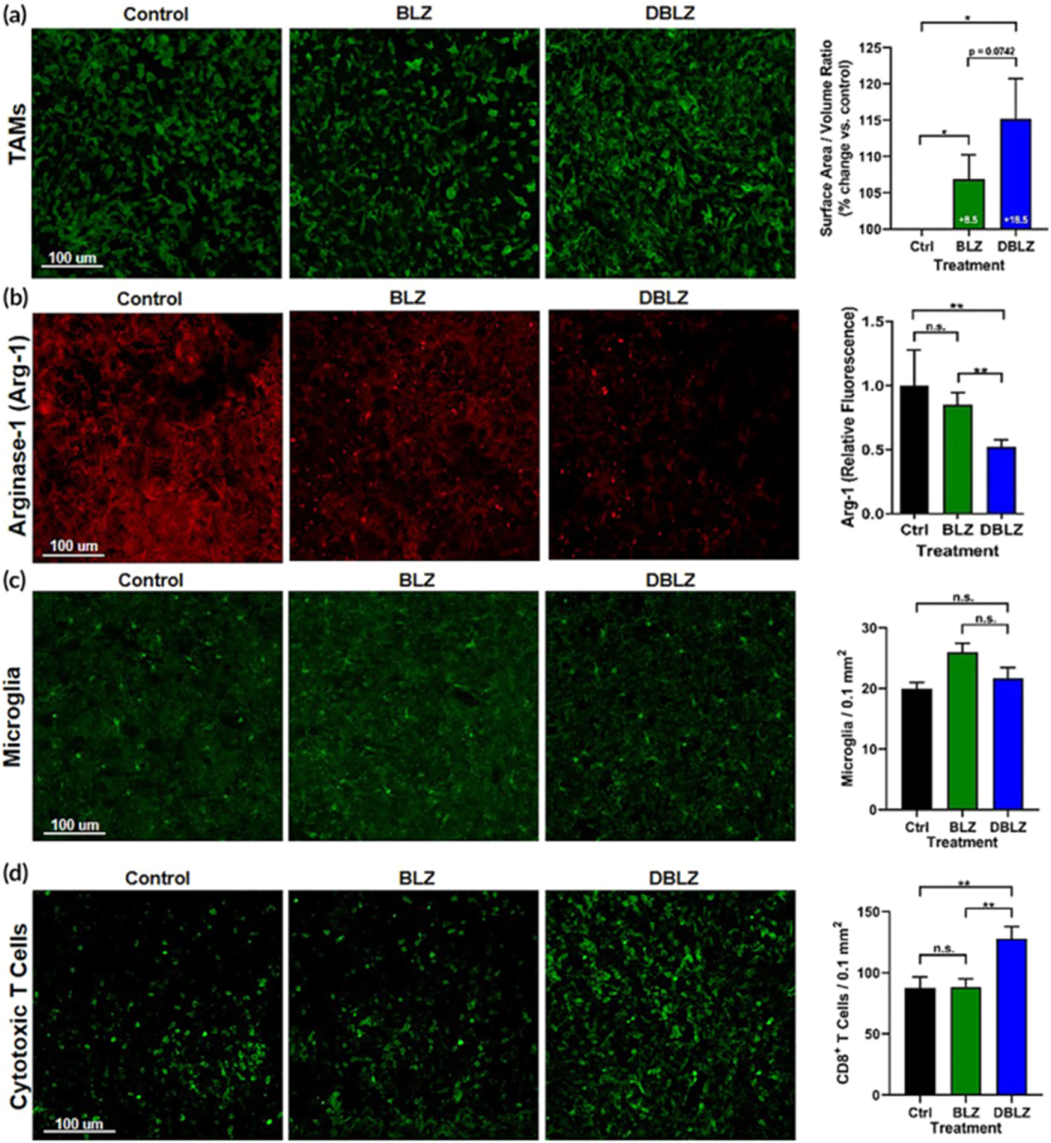
Targeted delivery of BLZ via DBLZ shifts TAMs from pro-tumor to antitumor polarization. (a) DBLZ increases TAM surface area-to-volume ratio (*p <. 05). (b) Arg-1 expression in TAMs decreases with DBLZ (**p <. 01). (c) Microglia presence is unchanged. (d) DBLZ enhances cytotoxic CD8+ T cell infiltration (*p <. 05). Error bars indicate mean ± SE, *N* = 10 animals, *n* = 3 images per group. Adapted with permission from.^134^ Copyright 2020 John Wiley & Sons Kevin Liaw.

In the study by Watson et al., the researchers discuss preclinical studies that investigate the influences of CSF1R inhibition in glioblastoma models, particularly focusing on the PDGFB-driven glioblastoma murine model. These studies employed a treatment regimen combining BLZ945 (a CSF1R inhibitor), galunisertib (GAL, a TGF-β inhibitor), and dexamethasone (DEX) to assess their impact on tumor progression and treatment-induced fibrosis. In these trials, mice were monitored biweekly using MRI to track tumor growth and recurrence. The findings suggested that the combination of BLZ945 with GAL and DEX significantly reduced treatment-induced fibrosis and several remaining tumor cells compared to other treatment regimens. Additionally, studies highlighted that the absence of BLZ945 led to rapid tumor progression and increased morbidity, underscoring the importance of CSF1R inhibition in managing glioblastoma. The findings from these preclinical studies suggest that targeting the fibrotic response alongside CSF1R inhibition could improve treatment efficacy and patient outcomes, paving the way for future clinical applications.^135^

Another study by Van Overmeire et al. was performed to study the roles of CSF-1 and GM-CSF signaling in the differentiation and functional polarization of TAMs from Ly6C^hi^ monocytes in various tumor models. It aimed to elucidate how these signaling pathways influence the abundance and phenotype of distinct TAM subsets, focusing on MHC-II^lo^ and MHC-II^hi^ populations. Muse models bearing 3LL-R tumors to assess the efficacy of CSF1R and GM-CSF1R signaling on TAM differentiation. It was observed that the CSF1R blockade significantly reduced the differentiation of Ly6C^hi^ monocytes into MHC-II^lo^ TAMs, shifting the balance toward MHC-II^hi^ TAMs and enhancing the antitumor immunity. Additionally, both these TAMs show increased capacity to stimulate CD4+ and CD8+ T cells, exhibiting a shift toward a more immunogenic profile. Conversely, GM-CSFR does not significantly affect TAM abundance or differentiation. Figure 5 illustrates the effects of α-CSF1R treatment on tumor-infiltrating monocytes. These findings focus on the critical role of CSF-1 signaling in antitumor responses, warranting further investigation into CSF1R-targeted therapies for cancer immunotherapy.^136^

**Figure 5:**
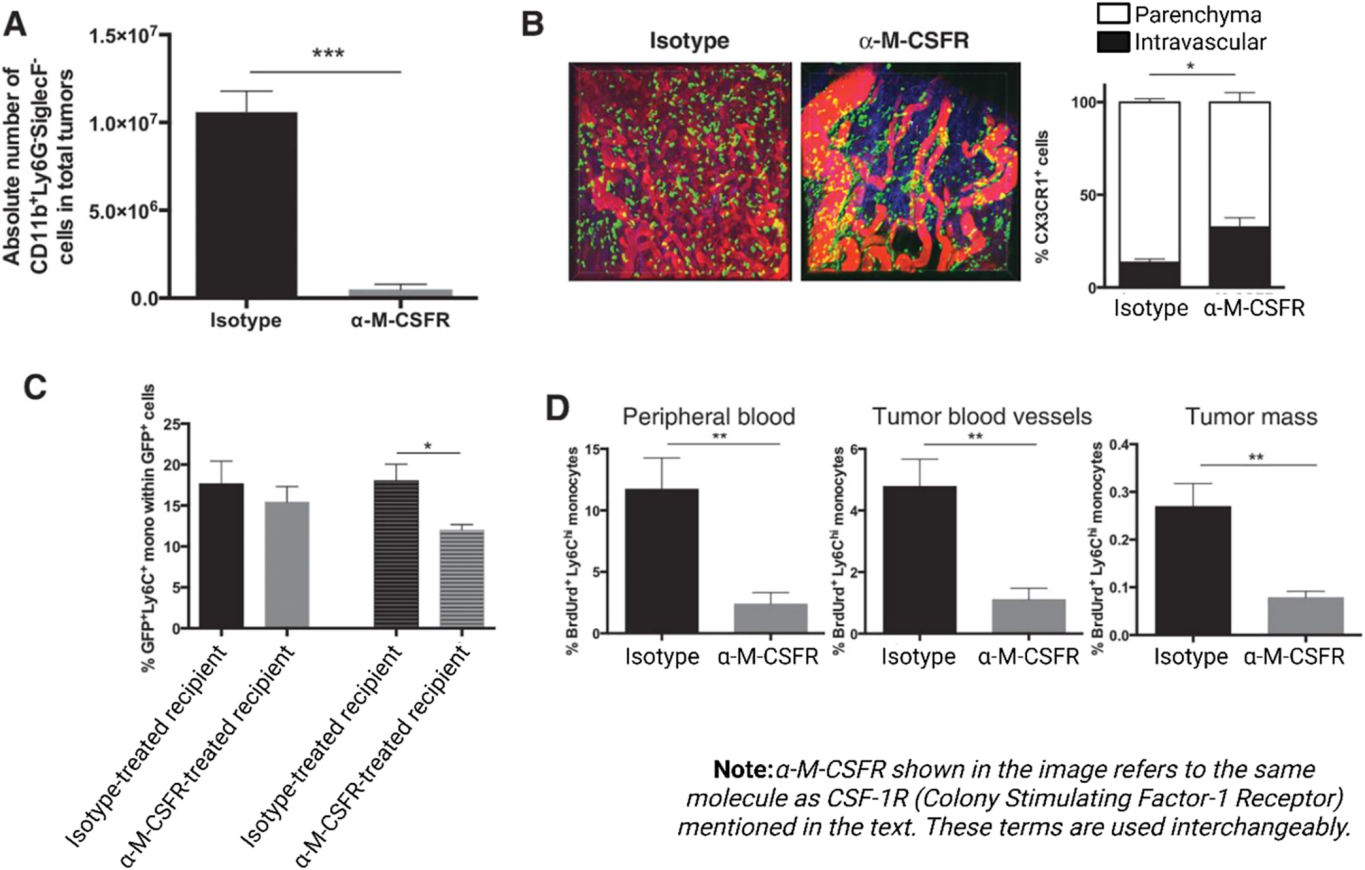
M-CSFR Modulates Tumor-Infiltrating Monocytes. (a) Quantification of CD11b+Ly6G–SiglecF– monocytes in 3LL-R tumor single-cell suspensions after treatment with isotype or α-M-CSFR antibodies from day 0 to day 14 (*n* ≥ 4, three independent experiments). (b) Intravital imaging of CX3CR1-GFP± reporter mice treated with isotype or α-M-CSFR antibody, analyzing the percentage of CX3CR1+ cells in tumor parenchyma and blood vessels (two fields per animal, two animals per group). (c) Assessment of M-CSFR blockade on monocyte recruitment via adoptive transfer of GFP+ bone marrow cells into 11-day-old 3LL-R tumor-bearing mice (*n* ≥ 4, three independent experiments). (d) Evaluation of M-CSFR blockade on monocyte proliferation in tumor-bearing mice receiving α-M-CSFR or isotype antibodies followed by BrdUrd injection, with results shown as mean ± SEM (*n* ≥ 4, three independent experiments). Statistical significance: *, *P* < 0.05; **, *P* < 0.01; ***, *P* < 0.001. Adapted with permission from.^136^ Copyright 2016 American Association for Cancer Research Eva Van Overmeire.

Stessin et al. aimed to investigate the role of the anti-CSF1R antibody AFS98 in inhibiting bone-marrow-derived macrophages and its effect on the therapeutic efficacy of combined stereotactic radiation therapy (SRT) and anti-PD-1 blockade in glioma bearing mice. There was a synergistic effect of the combination of SRT with anti-PD-1, which substantially improved survival rates due to the ability of SRT to enhance the influx of immune cells, especially CD8+ T-lymphocytes and M1 macrophages, which work together to mount a stronger antitumor immune response. Similarly, Figure 6 illustrates the differential expressions of CD86 and CD206 in macrophages, emphasizing the improved M1 polarization in the treatment group. This is essential as M1 macrophages are associated with antitumor activity, indicating that AFS98 may improve the efficiency of immunotherapy in glioma treatment.^137^

**Figure 6:**
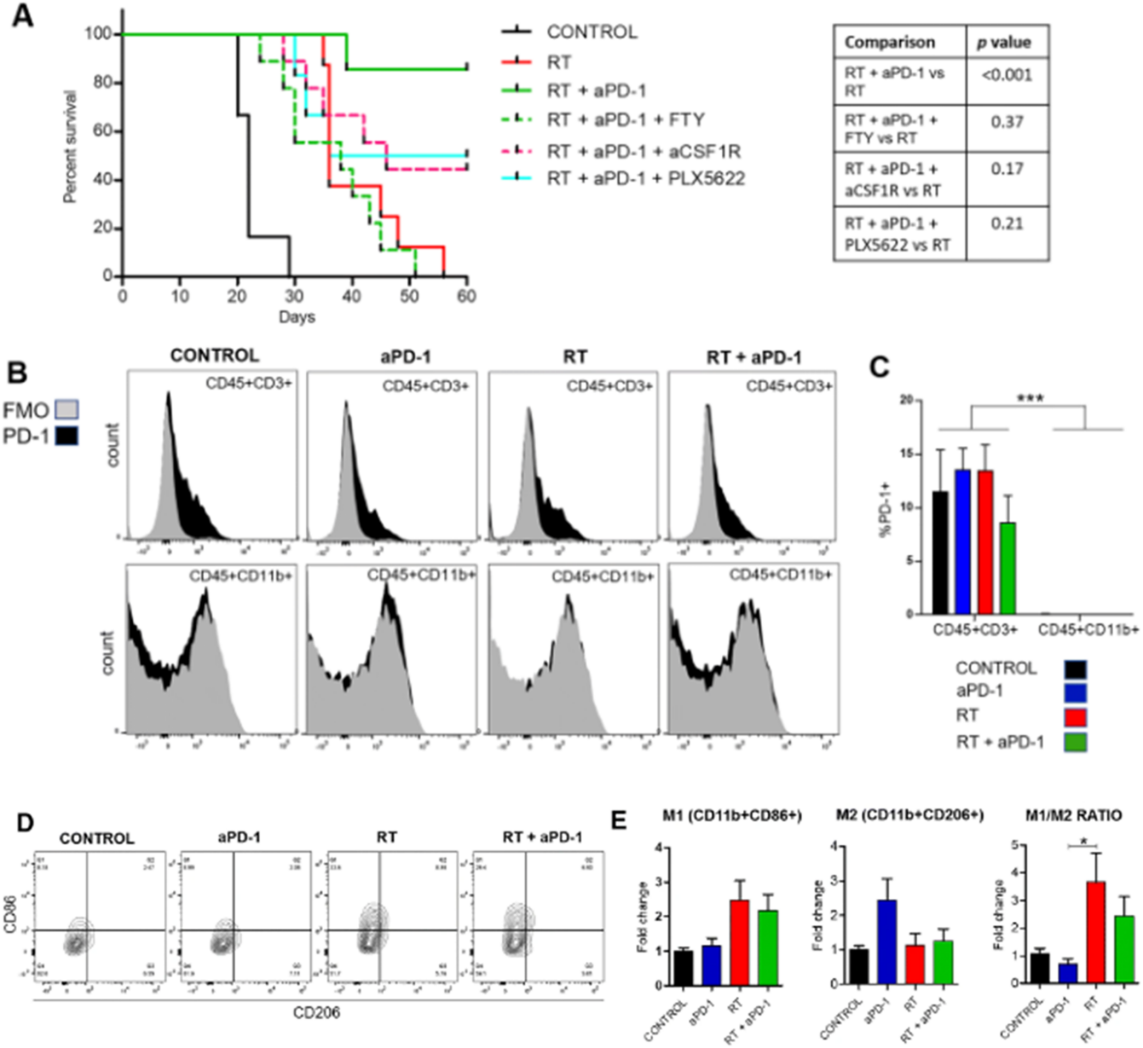
Immune Effector Cell Roles in Survival. (a) Kaplan–Meier survival curves showed decreased survival in CD8-depleted (FTY720), macrophage-depleted (anti-CSF1R), and microglia-depleted (PLX5622) groups. Survival differences were analyzed by the log-rank Mantel-Cox test. (b) Flow cytometry showed PD-1 expression on T cells (CD45+CD3+) and macrophages (CD45+CD11b+), with FMO controls. (c) PD-1 expression was significantly higher in treated groups (***P < 0.001 by two-way ANOVA). (d) The flow cytometry contour plots on day 15 postimplantation show the differential expression of CD86 and CD206 in CD45+CD11b + macrophages. (e) Percentages of M1-like (CD86+) and M2-like (CD206+) macrophages and the ratio of M1/M2 in the treatment groups. SRT induced a significant increase of M1 macrophages, *n* = 6 per group, *P < 0.05 and **P < 0.01 by ANOVA followed by Tukey’s post hoc. Adapted with permission from.^137^ Copyright 2020 Springer Nature Alexander M. Stessin.

Hu et al. performed the study to assess the efficacy of PLX5622 in depleting microglia and modulating TME in GBM models while focusing on its potential as a therapeutic agent in combination with temsirolimus. The study was carried out in female BALB/c nude mice, and assessment was done using IBA1 staining to quantify microglia depletion and evaluate tumor cell proliferation, angiogenesis, and macrophage polarization using various staining techniques. It was observed that PL5622 significantly reduced the amount of IBA1-positive microglia by approximately 18.14%, indicating its efficacy. The downregulation of M2-associated markers was also reported, suggesting a shift in macrophage polarization that could inhibit tumor progression. These findings are depicted in Figure 7, showing a comparison between IBA1 staining of treated and control groups, underlining the reduced presence of microglia in the PLX5622 treated group, correlating with decreased tumor cell proliferation and angiogenesis.^138^

**Figure 7:**
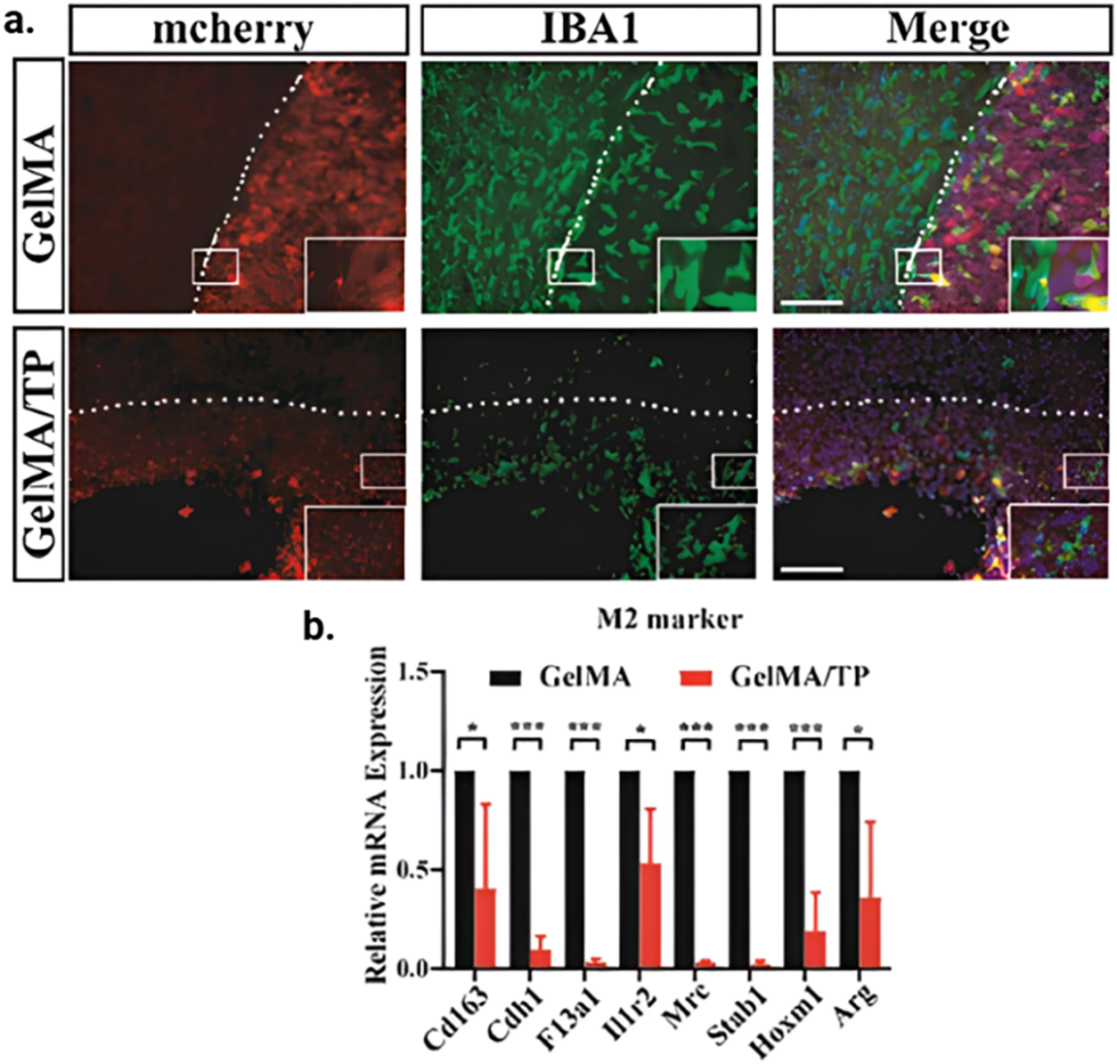
The mTOR pathway and TME modulation potencies of the intracavity-injected hydrogel system in brain tissue. (a) Representative IBA1 staining image in each group (scale bar = 100 μm). (b) Graph of the expression level of M2-associated genes in each group (*n* = 3 in each group, * *p* ≤ 0.05, *** *p* ≤ 0.001, Student’s *t* test). Adapted with permission from^138^ Copyright 2024 John Wiley & Sons Yang Hu.

Liu et al. performed preclinical studies wherein SYHA1813, a CSF1R inhibitor, was evaluated for its antitumor efficacy in both subcutaneous transplant models and intracranial glioma models using U251 and U87MG tumor cells in nude and NOD-SCID mice. The compound was administered orally, and results showed significant tumor growth inhibition, indicating its potent antiglioma activity. Furthermore, pharmacokinetic studies revealed that SYHA1813 effectively crosses the blood-brain barrier, achieving brain concentrations nearly equal to those in the bloodstream, which is crucial for treating central nervous system tumors. Additionally, SYHA1813 demonstrated the ability to reduce the infiltration of T-cell-suppressive TAMs, thereby improving the cytotoxic activity of CD8+ T cells within the TME. This immunomodulatory effect suggests that SYHA1813 could synergize with immune checkpoint therapies, providing a strong rationale for its clinical development in combination with other treatment modalities for glioblastoma. Overall, these findings underscore SYHA1813’s potential as a promising therapeutic agent against glioblastoma in preclinical settings.^139^

Another study by van der Wildt et al. discusses CSF1R inhibitors as a significant target for treating glioblastoma and other neuroinflammatory diseases. In this study, CSF1R inhibitors were radiolabeled and administered to mice to assess brain uptake and distribution. It was observed that there was a high brain uptake of the compound at various time points, ranging from 3.3 to 2.4% ID/g, indicating its efficient permeation through the BBB. Moreover, blocking studies have demonstrated that CSF1R inhibitors demonstrated selective binding to CSF1R, observed from the substantial difference in the brain uptake between baseline and blocking conditions. It was also inferred that approximately 80% of the tracer remained intact in brain homogenates at each time point, emphasizing the metabolic stability of the intervention. These observations state the potential of CSF1R inhibitors in animal models for CSF1R overexpression, especially in cases of brain tumors.^140^

The preclinical study by Zomer et al. investigated the effectiveness of CSF1R inhibition in treating GBM by targeting TAMs, which facilitate tumor growth and immune evasion. The results from preclinical studies demonstrate that treatment with the selective CSF1R inhibitor BLZ945 significantly reduced TAM populations within the TME, resulting in substantial tumor regression and a marked increase in survival rates for treated animal models. Specifically, the study reports that animals receiving BLZ945 demonstrated prolonged survival compared to control groups, highlighting the potential of CSF1R inhibition as a therapeutic approach for GBM. However, the findings also reveal that certain genetic alterations in gliomas can protect TAMs from depletion, suggesting that the effectiveness of this treatment may vary across different GBM subtypes and emphasizing the need for further research to understand the underlying mechanisms influencing TAM behavior and treatment response.^141^

The preclinical studies discussed in this section emphasize the substantial potential of CSF1R inhibition as a therapeutic strategy for GBM as discussed in Table 2. Across various animal models, targeting CSF1R demonstrated potential in reprograming TAMs, enhancing immune cell infiltration and overall survival outcomes. Furthermore, these studies have also demonstrated that CSF-QR inhibition can regulate the TME, reduce immunosuppressive responses, and possibly synergize with other therapeutic approaches. The results have concluded that these interventions can inhibit tumor growth and promote a more robust antitumor immune response, establishing the prognosis of GBM patients. The genetic heterogeneity of GBM and the protective role of certain mutations in TAMs limits efficacy across all subtypes and remains a challenge. While preclinical studies have shown potential, translating these results to human patients requires overcoming challenges such as effective brain delivery and immune modulation without off-target effects. Developing advanced drug delivery, exploring combination therapies with other treatments, and improving animal models to better comprehend human tumor diversity should be the main goals of future research on CSF1R inhibition in GBM.

## Clinical Trials

Clinical trials are critical for translating preclinical research into effective therapies, particularly for glioblastoma. Table 3 highlights key findings, including improved survival rates and reduced side effects from novel immunotherapies compared to standard treatments. These trials refine therapies, identify responsive patient subgroups, and set the stage for regulatory approvals. The table underscores their role in advancing personalized, safe, and effective care, emphasizing their transformative impact on patient outcomes.

**Table 3:** Clinical Trials Related to the CSF-1 and CSF1R in the Treatment of GBM

treatment	purpose	claim	status	clinical trials.gov identifier	refs
PLX3397 (oral)	Evaluate efficacy and safety in recurrent glioblastoma patients, focusing on 6-month progression-free survival (PFS6) and tumor tissue pharmacokinetics/pharmacodynamics	CSF1R inhibition reduces TAMs, potentially slowing GBM progression by altering TME	Phase II	NCT01349036	^142^
BLZ945 (oral CSF1R inhibitor) and PDR001	To evaluate safety, tolerability, pharmacokinetics (PK), and antitumor activity in advanced solid tumors. Phase I focuses on dose escalation to determine Maximum Tolerated Dose (MTD) and Recommended Phase 2 Dose (RP2D). Phase II assesses antitumor activity in glioblastoma patients.	BLZ945 targets CSF1R, potentially disrupting TAMs to enhance antitumor response. Combination with PDR001 (anti-PD-1) may improve immune activation	Phase I/II	NCT02829723	^143^

## Future Perspective

The future perspective for targeting CSF-1 and CSF1R in macrophage survival and function, particularly in glioblastoma treatment, is promising. As research continues to unveil intricate interaction between glioblastoma and the immune system, particularly the role of TAMs, a range of trends and innovations are emerging that could significantly enhance treatment outcomes for this aggressive form of cancer. One key trend is the increasing focus on personalized medicine approaches that leverage the distinct biological and molecular characteristics of individual tumors. By profiling the unique immune landscapes of glioblastomas, researchers aim to identify specific macrophage subtypes and their functional states that correlate with disease progression and response to treatments. Techniques, for instance, single-cell RNA sequencing and multiomics analyses are employed to map macrophage heterogeneity within the tumor microenvironment, providing insights into how CSF-1 and CSF1R influence macrophage behavior based on the tumor subtype. This data-driven approach will aid the development of targeted therapies that can repolarize macrophages from a tumor-promoting phenotype (M2) to a pro-inflammatory one (M1), thereby improving antitumor immunity.^144,145^

Another significant direction is exploring the role of combination therapies integrating CSF1R inhibitors with established treatment modalities, such as chemotherapy and immunotherapy. Preclinical studies have indicated that CSF1R blockade can inhibit tumor progression and improve the efficiency of present therapies by alleviating the immune suppression typically exerted by TAMs. Clinical trials are now investigating how well-tolerated CSF1/CSF1R inhibitors may boost the effectiveness of immune checkpoint inhibitors, suggesting a synergistic effect that could lead to better patient outcomes.^146^

Innovative therapeutic strategies are on the horizon, particularly concerning using small-molecule CSF1R inhibitors. Transformative candidates, such as PLX3397 and BLZ945, have shown promising results in preclinical models by effectively repolarizing TAMs and enhancing the immunogenicity of glioblastomas. The capability of inhibitors to modulate the TME while preserving necessary macrophage functions opens a new frontier in therapy. Moreover, the advent of nanotechnology for drug delivery represents another breakthrough. Nanoparticles designed to deliver CSF1R inhibitors directly to tumors could enhance the localized effects of treatment, reduce systemic side effects, and improve drug efficacy. These targeted delivery systems can be engineered to respond to specific tumor markers, ensuring a higher concentration of drugs at the site of action.^144^ Additionally, there is growing interest in dendritic cell vaccines that can exploit the reprogrammed TAMs resulting from CSF1R blockade. By using these reeducated macrophages as a potential adjuvant for vaccine therapies, researchers hope to improve T-cell responses against glioblastoma, thereby harnessing the complete power of the immune system in the fight against tumors.^147^

## Conclusion

The investigation into CSF-1 and CSF1R illuminates their crucial roles in macrophage biology and highlights their therapeutic potential in GBM. CSF-1 signaling is critical for macrophage survival, proliferation, and differentiation. Through its interaction with CSF1R, CSF-1 influences the fate of macrophages, guiding them toward M2 polarization, which is associated with tumor promotion and immune suppression. This duality makes CSF-1 and CSF1R integral components in determining macrophage functionality within the TME, where their balance can significantly affect tumor progression and patient outcomes. The potential for targeting the CSF1R pathway in glioblastoma treatment offers exciting prospects for enhancing therapeutic efficacy. Notably, preclinical studies utilizing CSF1R inhibitors like BLZ945 and PLX3397 show promise in not only reducing tumor recurrence but also in reprogramming TAMs from an M2-like pro-tumor state to antitumor M1-like state. This reprogramming can potentiate antitumor responses while preserving the beneficial functions of M1 macrophages, thereby enhancing overall treatment outcomes.

Future exploration should continue to explore the mechanisms underlying TAM behavior and their interactions with other immune cells in the TME. Investigations into combination therapies that integrate CSF1R inhibition with existing immunotherapies, such as immune checkpoint inhibitors, may prove invaluable in overcoming the challenges posed by the immunosuppressive nature of GBM. In conclusion, understanding and manipulating macrophage polarization through targeted CSF1R inhibition presents a promising strategy for treating glioblastoma. As research progresses, it is essential to develop precise, targeted therapies that account for individual tumor biology, thus improving treatment efficacy and patient survival. The overall significance lies in the ability to reorient macrophage functions within the TME, creating a more favorable immune environment that combats tumor growth and mitigates the risks associated with current treatment modalities. The journey from bench to bedside must focus on translating these insights into actionable therapies, improving the outlook for patients affected by this aggressive cancer.

## Abbreviation

AFS98: anti-fibroblast serum 98
AID: activation-induced cytidine deaminase
AKT: protein kinase B
AKT1: protein kinase B alpha
AKTs: protein kinase B isoforms
AMPK: AMP-activated protein kinase
ATF3: activating transcription factor 3
BBB: blood-brain barrier
BLZ: benzylidene derivative (context specific)
BNLZ: basic nuclear localization zipper
CAN: cancer antigen
CCL8: C–C motif chemokine ligand 8
CCR2: C–C chemokine receptor type 2
CD16/CD23/CD3/CD4/CD8: cluster of differentiation 16/23/3/4/8
CNS: central nervous system
CSF: colony stimulating factor
CSF-1: colony stimulating factor 1
CSF1R: colony stimulating factor 1 receptor
CT: computed tomography
DC: dendritic cell
DNA: deoxyribonucleic acid
ECM: extracellular matrix
EGFR: epidermal growth factor receptor
ELISA: enzyme-linked immunosorbent assay
FBS: fetal bovine serum
FDA: food and drug administration
GBM: glioblastoma multiforme
GTP: guanosine triphosphate
H&E: hematoxylin and eosin
HLA: human leukocyte antigen
IC50: half maximal inhibitory concentration
IFN: interferon
IFN-γ: interferon γ
IL/IL-10/IL-12/IL-4/IL-6: interleukin/10/12/4/6
IR: infrared
iNOS: inducible nitric oxide synthase
LPS: lipopolysaccharide
MAPK: mitogen-activated protein kinase
MCP: monocyte chemoattractant protein
MCP-1: monocyte chemoattractant protein 1
mRNA: messenger ribonucleic acid
MTT: 3-(4,5-dimethylthiazol-2-yl)-2,5-diphenyltetrazolium bromide
NF-κB: nuclear factor kappa-light-chain-enhancer of activated B cells
NK: natural killer
PBS: phosphate-buffered saline
PD-1: programmed cell death protein 1
PD-L1: programmed death-ligand 1
PET: positron emission tomography
PGE2: prostaglandin E2
pH: potential of hydrogen
PI3K: phosphoinositide 3-kinase
qRT-PCR: quantitative reverse transcription polymerase chain reaction
RNA: ribonucleic acid
ROS: reactive oxygen species
RPMI: roswell park memorial institute medium
siRNA: small interfering ribonucleic acid
STAT3: signal transducer and activator of transcription 3
TAM: tumor-associated macrophage
TGF-β: transforming growth factor β
TLR: toll-like receptor
TNF-α: tumor necrosis factor α
VEGF: vascular endothelial growth factor
